# Advocating hormonal treatment to prevent adult infertility in patients diagnosed with congenital undescended testes

**DOI:** 10.1590/S1677-5538.IBJU.20244.9902

**Published:** 2024-03-18

**Authors:** Faruk Hadziselimovic

**Affiliations:** 1 University of Basel Cryptorchidism Research Institute Liestal Switzerland University of Basel, Director of Cryptorchidism Research Institute, Kindermedizinisches Zentrum 4410 Liestal, Switzerland

**Keywords:** Hormonal treatment, Infertility, Cryptorchidism

## Abstract

In 2007 the Nordic group came to the following unanimous conclusions: In general, hormonal treatment is not recommended, considering the poor immediate results and the possible long-term adverse effects on spermatogenesis. Thus, surgery is to be preferred. However, defective mini puberty inducing insufficient gonadotropin secretion is one of the most common causes of nonobstructive azoospermia in men suffering from congenital isolated unilateral or bilateral cryptorchidism. The extent of alteration in the unilateral undescended testis correlate with the contralateral descended testis, indicating that unilateral cryptorchidism is a bilateral disease. Idiopathic central hypogonadism explains the phenomenon of defective mini puberty in otherwise healthy cryptorchid boys. We therefore recommend hormonal treatment for cryptorchid boys with defective mini puberty. Gonadotropin releasing hormone agonist (GnRHa) treatment following surgery to correct cryptorchidism restores mini puberty via endocrinological and transcriptional effects and prevents adult infertility in most cases. Several genes are important for central hypogonadotropic hypogonadism in mammals, including many that are transcribed in both the brain and testis. At the molecular level, there is no convincing evidence that heat shock is responsible for the observed pathological testicular changes. Thus, impaired transformation of gonocytes is not the result of temperature stress but rather a hormonal imbalance. Cryptorchidism should therefore be considered a serious andrological problem that cannot be successfully treated by early orchidopexy alone.

## THE PHYSIOLOGY OF MALE REPRODUCTION AND INFERTILITY

### The major goal of treating cryptorchidism.

The primary objective in treating cryptorchidism is to attain fertility. The estimated frequency of azoospermia in the general population is 0.4% (3/711) (1), with non-obstructive azoospermia occurring 25 times more frequently in unilateral and 80 times more frequently in bilateral cryptorchidism (2). Consequently, cryptorchidism stands as the most prevalent cause of azoospermia in men (3).

It is noteworthy that the incidence of azoospermia or severe oligospermia does not differ between patients who undergo surgery before and after the first year of age (p = 0.39, Fisher's Exact test; Feyles et al. 2014, as reviewed in reference (4). Therefore, severe infertility and azoospermia manifest in cryptorchidism regardless of the age at which treatment is administered (2, 5). As a result, early and ostensibly successful orchidopexy does not enhance fertility since it fails to address the underlying pathophysiological cause, namely defective mini-puberty (5).

During disrupted mini-puberty, inadequate LH secretion leads to diminished Leydig cell capacity and reduced testosterone levels, resulting in impaired Ad spermatogonia development and infertility (6). Moreover, it has been established that the differentiation of gonocytes into Ad spermatogonia is testosterone-dependent (7). Half of the patients with unilateral cryptorchidism and the majority with bilateral cryptorchidism fall into the high infertility risk (HIR) group (8). Consequently, establishing fertility is significantly reliant on a normal mini-puberty, essential for establishing a standard population of Ad spermatogonia (9, 10).

In 1975, pronounced Leydig cell atrophy, commencing in early infancy, was identified as evidence supporting endocrinopathy as an etiological factor in cryptorchidism (11). However, apart from a blunted testosterone response to human chorionic gonadotropins (HCG), no evidence supports altered steroidogenesis in cryptorchid testes before puberty (12). HIR cryptorchid boys exhibit low basal and stimulated gonadotropin plasma levels comparable to those in cases of hypogonadotropic hypogonadism (13, 14). Furthermore, numerous LH-RH tests have demonstrated a lower LH response to gonadotropin-releasing hormone (12). Thus, the cause of the diminished testosterone response appears to be at both the pituitary and hypothalamic levels.

### Is paternity a fertility indicator?

Lee and his co-authors conducted a retrospective review of medical records using a comprehensive questionnaire, determining that unilateral cryptorchid men exhibit a normal paternity rate (15). It is essential to highlight that this study did not rely on the results of testicular biopsies. Consequently, an evaluation of testicular tissue quality and the presence of Ad spermatogonia was absent. Patients were classified as cryptorchid solely based on the fact that they had undergone surgery. Notably, the unavoidable inclusion of low, intermediate, and high infertility risk patients, along with misdiagnosed cases of retractile testes, distorts the results. Therefore, relying solely on paternity as a fertility indicator is inadequate. More significantly, only a testicular biopsy can identify patients likely to be infertile, thus benefiting from hormonal therapy. This underscores that the rationale behind testicular biopsy is both diagnostic and therapeutic.

Furthermore, the justification for testicular biopsy extends to the detection of in situ carcinoma, occurring in 0.4% of the cases (16).

### The sperm count in long-term follow-up studies.

Approximately 47.5% of unilateral and 78% of bilateral cryptorchid males exhibit sperm concentrations within the infertility range according to WHO standards (17). In the HIR group of cryptorchid men, severely decreased sperm counts were observed, with no age-related differences, indicating that a successful orchidopexy is insufficient to prevent infertility and azoospermia development (10, 18).

In cases where differentiation into Ad spermatogonia had occurred (indicating functional mini-puberty), age-related differences in fertility outcomes were noted: the younger the unilateral cryptorchid boys were at surgery, the higher the adult sperm count. However, the difference in sperm count between boys younger than three years at the time of surgery (median; 156 × 106/ejaculate) and those older than eight years (mean; 87 × 106/ejaculate) is statistically significant but biologically irrelevant (10). Both groups displayed a total sperm count within the normal range.

A 20-year long-term prospective study initiated in 1985 in Philadelphia by the late John Duckett and Faruk Hadziselimovic yielded results in accordance with our previous study from 2005, emphasizing the importance of an intact hypothalamus-pituitary-testicular axis for normal fertility in cryptorchid men (17). Sperm concentrations correlated with the number of Ad spermatogonia found at the time of orchidopexy (p < 0.001). In the HIR group lacking Ad spermatogonia, 80% of males were oligospermic, and 20% displayed azoospermia (17). In patients with unilateral cryptorchidism, 70% of the scrotal testes showed varying degrees of impaired differentiation of Ad spermatogonia, indicating that cryptorchidism is a bilateral disease. Moreover, correlations between testicular histology and post-pubertal hormone levels confirmed various levels of gonadotropin deficiency in the majority of adult cryptorchid men. A crucial finding is that gonadotropin levels exhibit a more significant correlation with the presence or absence of Ad spermatogonia in both gonads than with unilateral or bilateral undescended testes (17).

Notably, in HIR patients, adequate treatment with low doses of GnRHa resulted in a normal sperm count in 86% of cases, and none developed azoospermia. This starkly contrasts with the results of the `surgery-only’ group, where not a single patient displayed a normal sperm count, and 20% were diagnosed with azoospermia (19).

RNA PROFILING TESTICULAR BIOPSIES YIELDS DATA THAT SUPPORT HORMONAL TREATMENT

Gene expression and function in the hypothalamus and pituitary gland

Significant differences in gene expression levels between testicular samples from high- and low-infertility risk groups were observed in various hypothalamic and pituitary genes. In 2016, we reported decreased expression of PROK2, CHD7, FGFR1, and SPRY4 in HIR testis from patients with impaired LH secretion (20). Mutations in these genes are associated with Kallmann syndrome (21). Notably, EGR4, a participant in regulating luteinizing hormone secretion, is virtually not expressed in HIR samples (22). Several genes involved in pituitary development and differentiation, such as ISL1, OTX2, PITX1, PITX2, GATA2, LHX2, LHX6, LHX8, and NHLH2, show a lower expression signal in HIR samples compared to LIR samples. ISL1, a paralog of LHX4, gained a new function during evolution (23). LHX4, encoding a transcription factor controlling pituitary gland development, belongs to a large protein family with the cysteine-rich zinc-binding LIM domain.

Moreover, deletion of OTX1 was found in six subjects with genitourinary defects, three of whom were diagnosed with cryptorchidism (24). It is worth noting that Otx2 heterozygous male mice display compromised fertility (reduced LH levels and testicular weight) due to a defect in the development, number, and migration of GnRH neurons (25). Since Otx1 and Otx2 have interchangeable functions, Otx2 could partially compensate for Otx1 deficiency in certain patients (26).

GnRHa treatment increases the expression of genes weakly transcribed in HIR samples, such as DLX1, DLX3, DLX6, EGR2, EGR3, ISL2, NR4A2, OTX1, OTX2, NHLH2, RUNX1, RUNX2, SIX2, SIX3, LEP, PCSK1, TAC3, and SOX family members. Interestingly, lower expression signals were found in HIR samples for long noncoding RNAs (lncRNAs) participating in epigenetic processes, including AIRN, FENDRR, XIST, and HOTAIR. These data support the hypothesis that hypogonadotropic hypogonadism in boys with altered mini-puberty is the consequence of a profoundly altered gene expression program involving protein-coding genes and lncRNAs (27, 28).

### Gene expression and function in testis

DMRTC2, PAX7, BRACHYURY/T, and TERT are associated with defective mini-puberty as they exhibit decreased expression in HIR samples and respond positively to GnRHa treatment (28). Notably, PAX7, EGR2, NRG1, and NRG3 appear to represent an alternative pathway activated by GnRHa, involved in regulating the differentiation of gonocytes into Ad spermatogonia. Additionally, differentially expressed genes like EGR2, ETV5, ID4, TSPAN8, and T are all regulated by FGF/GDNF signaling, while FOXO1, KIT, NANOS2, NRG1, NRG3, and PAX7 expression is regulated by retinoic acid (28). PAX7, BRACHYURY/T, EGR2, NRG1, and NRG3 are thereby linked to both FGF/GDNF and RA signaling (28). Interestingly, four genes localized on the male-specific Y chromosome - RBMY1B, RBMY1E, RBMY1J, and TSPY4 - show reduced mRNA levels in HIR samples and also positively respond to GnRHa treatment (29).

PRDM family members, such as PRDM1/BLIMP (30) and PRDM14 (31), play crucial roles in primordial germ cell specification, differentiation, and meiotic recombination in adult germ cells (32). PRDM9, differentially expressed in LIR versus HIR samples, is the only PRDM member found to be downregulated in HIR samples and stimulated by GnRHa treatment (33). PRDM9, a downstream effector of testosterone action, is related to testosterone-regulated cell proliferation in classical testosterone target tissues. Thus, PRDM9 is involved in establishing a normal Ad spermatogonia population, and its altered expression likely impacts male germ cell development in patients with cryptorchidism (33).

Impaired mini-puberty affects Sertoli cell development through both positive and negative regulation of morpho-regulatory and apoptotic genes. In contrast to germ cells, GnRHa treatment has a repressive effect on most Sertoli cell-specific genes, suggesting a cellular rearrangement in Sertoli cells. It is proposed that a gonadotropin-dependent increase in FASLG and GDNF expression drives Sertoli cell proliferation and germ cell self-renewal, thereby stimulating the transition of gonocytes to Ad spermatogonia. RNA-profiling experiments reveal novel testosterone-dependent genes, providing valuable insights into the transcriptional response to both defective mini-puberty and curative GnRHa treatment (34). In conclusion, EGR4 and PITX1, controlled by PROK2/CHD7/FGFR1/SPRY4, are involved in LH deficiency, affecting germ cell transitional effectors such as FGFR3, FGF9, NANOS2, NANOS3, SOHLH1, and SOHLH2. GnRHa activates alternative pathways comprising EGR2, EGR3, NHLH2, TAC1, TAC3, PROP1, and LEP, important for LH secretion, and DMRTC2, T, PAX7, TERT, NRG1, NRG3, RBMY1B, RBMY1E, and RBMY1J, involved in the differentiation of gonocytes into Ad spermatogonia (20, 28).

### Temperature or transposons – what are the critical factors for fertility?

At the molecular level, there is increasing evidence that challenges the notion of heat shock being predominantly responsible for the observed pathological testicular changes in the prepubertal testis (35). No differences in the expression of heat shock protein, endoplasmic reticulum, and heat factor genes were observed between undescended and descended testes (35). Contrary to the assumption that temperature-dependent effects on cryptorchid gonads damage undescended testes before sexual maturation is complete, recent evidence aligns with the idea that germ cell loss, resulting in infertility in cryptorchidism, is a consequence of alterations in the Piwi pathway and the derepression of transposons (36).

Several members of the Tudor gene family, as well as members of the DEAD-box RNA helicase family, and GTSF1, MEAL, and MOV10L1, were found to exhibit significantly lower RNA signals in testicular samples from HIR patients (25, 36). Patients from the low infertility risk (LIR) group consistently displayed stronger staining for GTSF1 and PIWIL4 and weaker staining for the L1 transposon compared to HIR samples (36). These findings provide initial evidence consistent with the idea that infertility in cryptorchidism is a consequence of alterations in the Piwi pathway and transposon derepression induced by the impaired function of mini-puberty.

Abnormal gametogenesis results from disturbed PIWIL biogenesis (involving four PIWIL genes) and insufficient ASZ1, FOXA1, and CFTR functions. Importantly, curative GnRHa treatment stimulates the expression of several genes involved in pituitary development and differentiation, neuronal development, and testosterone synthesis pathways (37). Thus, intact testosterone secretion and the function of P-bodies during mini-puberty contribute to the establishment of male-specific DNA methylation pathways.

### Is there a role for hormonal treatment in the epididymal-testicular descent prior to surgery?

The developing gonadotropin-releasing hormone (GnRH) system is crucial for epididymal-testicular descent and is sensitive to reduced fibroblast growth factor (FGF) signaling. Our understanding of the impact of FGFR1 in the process of epididymal-testicular descent has recently advanced. In later stages of embryonic development, the undifferentiated epididymal mesenchyme becomes a specific domain for FGFR1 expression (38). Individuals with syndromic crypto-epididymis and those with isolated non-descent of the epididymal-testicular unit often exhibit disturbances in FGF, FGFR1, and/or genes regulating the hypothalamic-pituitary-gonadal axis (38). However, the mechanisms underlying FGF dysregulation may vary among different syndromes.

The primary reason for not recommending hormonal treatment for the undescended epididymal-testicular union is purportedly the low success rate, reported as only 20% (39). This statement is misleading because it does not consider the distribution of the positions of the epididymal-testicular unit before treatment. Furthermore, studies cited as evidence against hormonal treatment lack critical extended follow-up examinations. One of the initial long-term follow-up studies demonstrated that four years after successful hormonal treatment, 65% of the testes remained descended (40). Notably, Höcht et al. published a randomized study with LH-RH or surgery groups, including 60 cryptorchid boys aged two to nine years (41, 42). All patients randomized for surgery treatment alone displayed histological changes compatible with cryptorchidism, indicating that only undescended and not retractile testes were treated. LH-RH treatment was successful in 59% of the patients (42). Nine years after treatment, 52% of testes remained descended (43). Thus, LH-RH treatment is effective in achieving the permanent descent of true cryptorchid testes. Notably, the highest success was achieved when testes were localized in the pre-scrotal position (Table-1).

**Table:** 

**1. LH-RH 1.2 mg/day for 28 days**.
-no descent
**2. 500 IU HCG/week for three weeks.**
-no descent
**3. Orchidopexy & testicular biopsy.**
-boys with <0.05 Ad spermatogonia/tubule
**4. GnRHa 10 µg intra-nasal on alternate days during six months.**

**Table 1:** Descent rate of the epididymal-testicular unit from the pre-scrotal position treated with gonadotropin-releasing hormone.

Author(s)	n	% success
deMuinck Keizer-Schrama et al. (44)	6/9	66
Borkenstein and Zobel (45)	5/9	55
Hagberg and Westphal (46)	8/17	47
Höcht (42)	3/4	75
Bica and Hadziselimovic (47)	6/11	54.5
**Total**	**28/50**	**56**

### A four-step treatment of prepubertal patients to prevent cryptorchidism-dependent infertility.

## CONCLUSION AND RECOMMENDATIONS

Abnormal germ cell development in cryptorchidism is not a congenital dysgenesis but rather an endocrinopathy, preceded by hormonal imbalance and perturbation of germ cell-specific gene expression during abrogated mini-puberty. GnRHa treatment of HIR (high infertility risk) patients induces a broad transcriptional response, encompassing protein-coding genes involved in pituitary development, the hypothalamic-pituitary-gonadal axis, and testosterone synthesis. Adequate treatment with low doses of GnRHa resulted in 86% of men displaying a normal sperm count, and notably, not a single patient presented with azoospermia.

Since abnormal mini-puberty is responsible for the development of infertility in cryptorchidism, post-surgical hormonal treatment is strongly recommended for the high infertility and azoospermia risk group of cryptorchid boys who underwent successful early orchidopexy. Furthermore, hormonal treatment to achieve epididymal-testicular descent as the primary choice of treatment for cryptorchidism has a long tradition in Europe. It eliminates the need for subsequent surgery, and in cases of non-responders, it facilitates orchidopexy, contributing to a reduced incidence of unilateral and the more serious bilateral complete post-surgical testicular atrophy. Therefore, the current and optimal therapeutic choice involves two steps of hormonal treatment..

## References

[1] Itoh N, Kayama F, Tatsuki TJ, Tsukamoto T. (2001). Have sperm counts deteriorated over the past 20 years in healthy, young Japanese men? Results from the Sapporo area. J Androl.

[2] Hadziselimovic F, Hadziselimovic NO, Demougin P, Oakeley EJ (2011). Testicular gene expression in cryptorchid boys at risk of azoospermia. Sex Dev.

[3] Fedder J, Crüger D, Oestergaard B, Petersen GB (2004). Etiology of azoospermia in 100 consecutive nonvasectomized men. Fertil Steril.

[4] Hadziselimovic F. (2017). On the descent of the epididymo-testicular unit, cryptorchidism, and prevention of infertility. Basic Clin Androl.

[5] Hadziselimovic F, Herzog B. (2001). The importance of both an early orchidopexy and germ cell maturation for fertility. Lancet.

[6] Hadziselimovic F, Zivkovic D, Bica DT, Emmons LR (2005). The importance of mini-puberty for fertility in cryptorchidism. J Urol.

[7] Zivkovic D, Bica DG, Hadziselimovic F. (2006). Effects of hormonal treatment on the contralateral descended testis in unilateral cryptorchidism. J Pediatr Urol.

[8] Bilius V, Verkauskas G, Dasevicius D, Kazlauskas V, Malcius D, Hadziselimovic F. (2015). Incidence of High Infertility Risk among Unilateral Cryptorchid Boys. Urol Int.

[9] Hadziselimović F, Thommen L, Girard J, Herzog B. (1986). The significance of postnatal gonadotropin surge for testicular development in normal and cryptorchid testes. J Urol.

[10] Hadziselimovic F, Emmons LR, Buser MW (2004). A diminished postnatal surge of Ad spermatogonia in cryptorchid infants is additional evidence for hypogonadotropic hypogonadism. Swiss Med Wkly.

[11] Hadziselimovic F, Herzog B, Seguchi H. (1975). Surgical correction of cryptorchism at 2 years: electron microscopic and morphometric investigations. J Pediatr Surg.

[12] Jockenhovel F, Swerdloff RS, Abney TO, Keel BA (1989). Alterations in steroidogenic capacity of Leydig cells in cryptorchid testis. The Cryptorchid testis.

[13] Hadziselimovic F, Herzog B, Girard J, Bierich JR, Giarola A (1979). Lack of germ cells and endocrinology in cryptorchid boys from one to six years of life. Cryptorchidism.

[14] Verkauskas G, Malcius D, Eidukaite A, Vilimas J, Dasevicius D, Bilius V (2016). Prospective study of histological and endocrine parameters of gonadal function in boys with cryptorchidism. J Pediatr Urol.

[15] Lee PA, O’Leary LA, Songer NJ, Coughlin MT, Bellinger MF, LaPorte RE. (1996). Paternity after unilateral cryptorchidism: a controlled study. Pediatrics.

[16] Hadziselimović F, Herzog B, Emmons LR (1997). The incidence of seminoma and expression of cell adhesion molecule CD44 in cryptorchid boys and infertile men. J Urol.

[17] Hadziselimovic F, Hoecht B. (2008). Testicular histology related to fertility outcome and postpubertal hormone status in cryptorchidism. Klin Padiatr.

[18] Hadziselimovic F, Hocht B, Herzog B, Buser MW (2007). Infertility in cryptorchidism is linked to the stage of germ cell development at orchidopexy. Horm Res.

[19] Hadziselimovic F. (2008). Successful treatment of unilateral cryptorchid boys risking infertility with LH-RH analogue. Int Braz J Urol.

[20] Hadziselimovic F, Gegenschatz-Schmid K, Verkauskas G, Docampo-Garcia MJ, Demougin P, Bilius V (2016). Gene Expression Changes Underlying Idiopathic Central Hypogonadism in Cryptorchidism with Defective Mini-Puberty. Sex Dev.

[21] Valdes-Socin H, Rubio Almanza M, Tomé Fernández-Ladreda M, Debray FG, Bours V, Beckers A. (2014). Reproduction, smell, and neurodevelopmental disorders: genetic defects in different hypogonadotropic hypogonadal syndromes. Front Endocrinol (Lausanne).

[22] Hadziselimovic F, Hadziselimovic NO, Demougin P, Krey G, Hoecht B, Oakeley EJ (2009). EGR4 is a master gene responsible for fertility in cryptorchidism. Sex Dev.

[23] Hadziselimovic F, Gegenschatz-Schmid K, Verkauskas G, Docampo-Garcia MJ, Demougin P, Bilius V (2016). Gene Expression Changes Underlying Idiopathic Central Hypogonadism in Cryptorchidism with Defective Mini-Puberty. Sex Dev.

[24] Jorgez CJ, Rosenfeld JA, Wilken NR, Vangapandu HV, Sahin A, Pham D (2014). Genitourinary defects associated with genomic deletions in 2p15 encompassing OTX1. PLoS One.

[25] Larder R, Kimura I, Meadows J, Clark DD, Mayo S, Mellon PL (2013). Gene dosage of Otx2 is important for fertility in male mice. Mol Cell Endocrinol.

[26] Acampora D, Avantaggiato V, Tuorto F, Barone P, Perera M, Choo D (1999). Differential transcriptional control as the major molecular event in generating Otx1-/- and Otx2-/- divergent phenotypes. Development.

[27] Hadziselimovic F, Gegenschatz-Schmid K, Verkauskas G, Demougin P, Bilius V, Dasevicius D (2017). GnRHa Treatment of Cryptorchid Boys Affects Genes Involved in Hormonal Control of the HPG Axis and Fertility. Sex Dev.

[28] Gegenschatz-Schmid K, Verkauskas G, Demougin P, Bilius V, Dasevicius D, Stadler MB (2017). DMRTC2, PAX7, BRACHYURY/T and TERT Are Implicated in Male Germ Cell Development Following Curative Hormone Treatment for Cryptorchidism-Induced Infertility. Genes (Basel).

[29] Gegenschatz-Schmid K, Verkauskas G, Stadler MB, Hadziselimovic F. (2019). Genes located in Y-chromosomal regions important for male fertility show altered transcript levels in cryptorchidism and respond to curative hormone treatment. Basic Clin Androl.

[30] Kobayashi T, Zhang H, Tang WWC, Irie N, Withey S, Klisch D (2017). Principles of early human development and germ cell program from conserved model systems. Nature.

[31] Shirane K, Kurimoto K, Yabuta Y, Yamaji M, Satoh J, Ito S (2016). Global Landscape and Regulatory Principles of DNA Methylation Reprogramming for Germ Cell Specification by Mouse Pluripotent Stem Cells. Dev Cell.

[32] Smagulova F, Brick K, Pu Y, Camerini-Otero RD, Petukhova GV (2016). Erratum: The evolutionary turnover of recombination hot spots contributes to speciation in mice. Genes Dev.

[33] Hadziselimovic F, Cathomas G, Verkauskas G, Dasevicius D, Stadler MB (2018). PRDM Histone Methyltransferase mRNA Levels Increase in Response to Curative Hormone Treatment for Cryptorchidism-Dependent Male Infertility. Genes (Basel).

[34] Gegenschatz-Schmid K, Verkauskas G, Demougin P, Bilius V, Dasevicius D, Stadler MB (2018). Curative GnRHa treatment has an unexpected repressive effect on Sertoli cell specific genes. Basic Clin Androl.

[35] Hadziselimovic F. (2022). Temperature is not a major factor in the differentiation of gonocytes into ad spermatogonia and fertility outcome in congenitally cryptorchid boys. Basic Clin Androl.

[36] Hadziselimovic F, Hadziselimovic NO, Demougin P, Krey G, Oakeley E. (2015). Piwi-pathway alteration induces LINE-1 transposon derepression and infertility development in cryptorchidism. Sex Dev.

[37] Hadziselimovic F, Verkauskas G, Stadler M. (2022). A novel role for CFTR interaction with LH and FGF in azoospermia and epididymal maldevelopment caused by cryptorchidism. Basic Clin Androl.

[38] Hadziselimovic F. (2016). Involvement of Fibroblast Growth Factors and Their Receptors in Epididymo-Testicular Descent and Maldescent. Mol Syndromol.

[39] Ritzén EM, Bergh A, Bjerknes R, Christiansen P, Cortes D, Haugen SE (2007). Nordic consensus on treatment of undescended testes. Acta Paediatr.

[40] Hadziselimović F, Girard J, Herzog B. (1984). 4 Jahre Erfahrung mit der hormonellen kombinierten Behandlung des Kryptorchismus [4 years’ experience with combined hormonal treatment of cryptorchism]. Z Kinderchir.

[41] Höcht B. (1983). Zur Therapie des präpuberalen Maldeszensus. Klinische Erfahrungen mit der LH-RH-Behandlung [Therapy of prepubertal cryptorchism. Clinical experiences with LH-RH treatment]. Fortschr Med.

[42] Höcht B. (1987). LH-RH treatment for cryptorchidism. Randomized study and 10-year follow-up results. Eur J Pediatr.

[43] Waldschmidt J, Doede T, Vygen I. (1993). The results of 9 years of experience with a combined treatment with LH-RH and HCG for cryptorchidism. Eur J Pediatr.

[44] deMuinck Keizer-Schrama SM, Hazebroek FW, Matroos AW, Drop SL, Molenaar JC, Visser HK (1986). Double-blind, placebo-controlled study of luteinising-hormone-releasing-hormone nasal spray in treatment of undescended testes. Lancet.

[45] Borkenstein M, Zobel V. (1985). Behandlung des Maldescensus testis mit LHRH-Nasalspray [Treatment of cryptorchism with LHRH nasal spray]. Wien Klin Wochenschr.

[46] Hagberg S, Westphal O. (1987). Results of combined hormonal and surgical treatment for undescended testis in boys under 3 years of age. A randomized study. Eur J Pediatr.

[47] Bica DT, Hadziselimovic F. (1992). Buserelin treatment of cryptorchidism: a randomized, double-blind, placebo-controlled study. J Urol.

[48] Bica DT, Hadziselimovic F. (1993). The behavior of epididymis, processus vaginalis and testicular descent in cryptorchid boys treated with buserelin. Eur J Pediatr.

